# Expression of Plant Sweet Protein Brazzein in the Milk of Transgenic Mice

**DOI:** 10.1371/journal.pone.0076769

**Published:** 2013-10-14

**Authors:** Sen Yan, Hong Song, Daxin Pang, Qingjian Zou, Li Li, Quanmei Yan, Nana Fan, Xiangjie Zhao, Hao Yu, Zhanjun Li, Haijun Wang, Fei Gao, Hongsheng Ouyang, Liangxue Lai

**Affiliations:** 1 Jilin Provincial Key Laboratory of Animal Embryo Engineering, College of Animal Science, Jilin University, Changchun, China; 2 Key Laboratory of Regenerative Biology, South China Institute for Stem Cell Biology and Regenerative Medicine, Guangzhou Institutes of Biomedicine and Health, Chinese Academy of Sciences, Guangzhou, China; Emory University, United States of America

## Abstract

Sugar, the most popular sweetener, is essential in daily food. However, excessive sugar intake has been associated with several lifestyle-related diseases. Finding healthier and more economical alternatives to sugars and artificial sweeteners has received increasing attention to fulfill the growing demand. Brazzein, which comes from the pulp of the edible fruit of the African plant *Pentadiplandra brazzeana* Baill, is a protein that is 2,000 times sweeter than sucrose by weight. Here we report the production of transgenic mice that carry the optimized brazzein gene driven by the goat Beta-casein promoter, which specifically directs gene expression in the mammary glands. Using western blot analysis and immunohistochemistry, we confirmed that brazzein could be efficiently expressed in mammalian milk, while retaining its sweetness. This study presents the possibility of producing plant protein–sweetened milk from large animals such as cattle and goats.

## Introduction

Palatal disposition towards sweet food is common to many animals including humans. However, excessive sucrose intake has been attributed to several lifestyle-related diseases, such as dental caries, obesity, and diabetes mellitus. The demand for natural and healthy low-calorie sweeteners is increasing. Six sweet proteins have been described as: thaumatin, monellin, mabinlin, pentadin, brazzein, and curculin [1]. Of these six proteins, brazzein has the smallest molecular weight and the highest heat resistance as well as good solubility. It comes from the West African plant *Pentadiplandra brazzeana* Baill and is distributed in the pulp of the fruit, which turns from green to red during ripening. Brazzein is intrinsically 2,000 times sweeter than 2% sucrose solution. The sweetness of brazzein persists even after incubation at 80°C for 4 hours [2]. The brazzein content of ripe fruits is approximately 0.2% to 0.05% by weight [3].

Unfortunately, the commercial production of brazzein and other sweet proteins has been limited because they come from tropical plants. Large-scale brazzein production will probably require protein expression in a heterologous system through genetic engineering. Sweet proteins have been engineered in *Escherichia coli* [4], *Lactococcus lactis* [5], and *Pichia pastoris* [6]. In 2005, brazzein production in transgenic maize yielded a large amount of the recombinant brazzein [7]. However, the protein needs to be isolated and purified before it can be used as an additive. Moreover, the protein tags of the brazzein produced from bacteria and yeast need to be cleaved to activate its sweetness [4],[8]. Milk carries a large quantity of protein and provides a safe, abundant, and easily obtainable source of raw materials. Transgenic manipulation of mammary glands has no detectable effects on the animal and no change of the contents of the animal milk [9]. Thus, the mammary glands of large domestic animals such as goat and cattle have been used as bioreactors to the large-scale production of valuable recombinant proteins. A previous study successfully expressed functional Δ12 fatty acid desaturase from spinach in transgenic pigs, which indicates that plant proteins can be expressed in mammalian cells [10].

In our study, we introduced the brazzein transgene into mice to produce brazzein in the mammary glands. We chose mouse mammary glands first as a model to verify the feasibility of our approach. We produced transgenic mice that carry the optimized brazzein transgene driven by the goat Beta-casein promoter, which specifically directs gene expression in milk. Using western blotting, immunohistochecmistry and sweetness intensity test, we show that brazzein is efficiently expressed in mammalian milk while retaining its sweetness.

## Materials and Methods

### Ethics Statement

The animal experiment facilities were approved by the Department of Science and Technology of Guangdong Province [approval ID SYXK (Guangdong) 2005-0063] and complied with the guidelines of the Animal Care Committee, Guangzhou Institutes of Biomedicine and Health, Chinese Academy of Sciences (Animal Welfare Assurance #A5748-01). All surgical procedures were performed under anesthesia, and all efforts were made to minimize animal suffering.

### Synthesize and Optimize of the Brazzein Gene

The brazzein gene was artificially synthesized through overlap extension PCR of 12 oligonucleotide fragments. The two oligonucleotides primers used (BrFF1∶5′-GAATTCTCGAGGCCATGAAGGTCCTGATCCTC-3′; BrSR2∶5′-GAATTCTCGAGTCAGTACTCGCAATAGTC-3′) contained *Xho*I sites at the 5′-terminal end to allow subcloning of the brazzein gene into pBC1 vector (Invitrogen, Carlsbad, CA, USA). The first GAG, which encodes glutamic acid, was deleted from brazzein gene sequence, the 29th codon was modified from GAC (aspartic acid) into AAG (lysine), and the 41th codon was changed from GAG (glutamic acid) into AAG (lysine), as described in a previous study [11]. The codons in the brazzein gene were further optimized for expression in animal cells. The synthesized brazzein gene fragment was fused with a bovine Beta-casein signaling peptide coding region, a Kozak translation initiation sequence with a start codon for translation initiation, and a stop codon for terminating translation [12]. The optimized brazzein gene sequence is as follows: GCC (Kozak sequence)ATGAA GGTCCTCATC CTTGCCTGCC TGGTGGCTCT GGCCCTTGCA (goat Beta-casein signal sequence) GAC AAG TGC AAG AAG GTG TAC GAG AAC TAC CCC GTG AGC AAG TGC CAG CTG GCC AAC CAG TGC AAC TAC GAC TGC AAG CTG AAG AAG CAC GCC AGG AGC GGC GAG TGC TTC TAC GAC AAG AAG AGG AAC CTG CAG TGC ATC TGC GAC TAC TGC GAG TAC TGA (Optimized brazzein gene sequence).

### Construction of the Universal and Mammary Gland-specific Brazzein Expression Vectors

**Figure 1:**
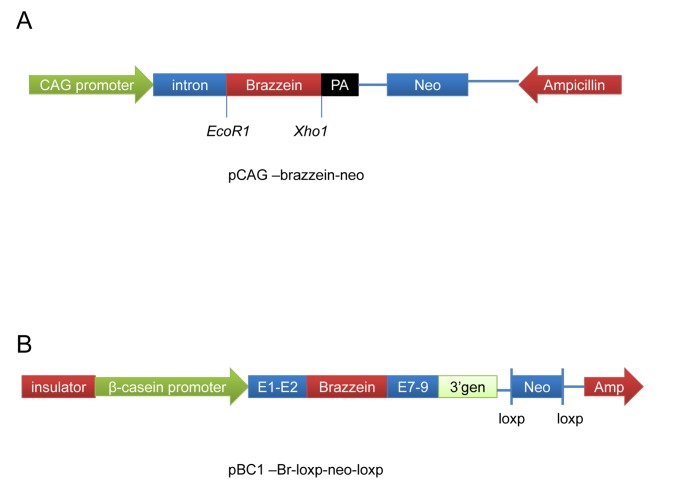
Schematic of pCAG–Brazzein-neo and pBC1-Br-loxP-neo-loxP. (A) Brazzein was directionally cloned into the EcoRI and XhoI sites of the pCAG vector. (B) Brazzein was cloned into the XhoI site of the pBC1 vector. The resulting constructs are composed of the following genetic elements: globin insulator, goat Beta-casein promoter, goat Beta-casein exon 1, intron 1, and part of exon 2 (before initial goat Beta-casein codon); brazzein, bovine Beta-casein signal peptide gene, and an optimized brazzein coding region; casein E7–E9, goat Beta-casein exon 7, intron 7, exon 8, intron 8, and exon 9; casein 3′ genomic DNA, and goat Beta-casein 3′ genomic region.

The PCR product was cloned into the pMD18-T simple vector (TaKaRa, Madison, Japan) and confirmed by sequencing. The brazzein gene fragment was digested with *EcoR*I and *Xho*I, and then inserted into the pCAG-neo vector to create the universal expression vector pCAG-Br-neo (Fig. 1A). To construct a mammary gland–specific expression vector, the Brazzein PCR product was digested with *Xho*I (TaKaRa, Dalian, China) and was cloned into pBC1 vector (Invitrogen, Carlsbad, CA, USA), which contains the goat Beta-casein promoter. A selectable loxP-neo-loxP cassette was cloned into the *Not*I site of the pBC1 vector. The resulting vector was designated as pBC1-Br-loxP-neo-loxP (Fig. 1B).

### Establishment of Stable Transgenic mES Cell Lines with Brazzein Transgene

Mouse embryonic stem (mES) cells isolated from a mouse (♂ Oct4 promoter-GFP×♀ 129) [13] was cultured on mitomycin-treated mouse embryonic fibroblasts (as feeder cells). The cells were grown in KO DMEM (Gibco) supplemented with 15% (v/v) fetal bovine serum (Gibco), 1% non-essential amino acids (Gibco), 1% glutamine (Gibco), 0.1 mM Beta-mercaptoethanol (Gibco), and 1000 units/mL leukemia inhibitory factor (ESGRO) at 37°C and 5% CO_2_. The mES cells were transfected with linearized pBC1-Br-loxP-neo-loxP vector using Lipofectamine 2000 transfection reagent (Invitrogen, Carlsbad, CA, USA) following the manufacturer’s protocol. The cells were diluted 1∶20 in fresh growth medium 24 h after transfection and then cultured in selection medium containing 300 ug/mL of G418 (Merck, CAS#108321-42-2, Germany) for 6 days. The surviving cell colonies were picked and maintained in 300 ug/mL G418. Positive transfected clones were determined by PCR with the oligonucleotide primers BR (Forward): 5′GCAGAAAACATCTGAATGGCTGGC-3′ and BR (Reverse): 5′CACTCGCCAGACCGAGCGTG-3′. Transfected mES cell lines were chosen to generate chimeric mice. Before transplantation, mES cells were collected, washed in PBS (pH 7.3), and centrifuged for 5 min at 300×g. The feeders were thoroughly removed through differential speed adherence. After aspirating the mES cells in the supernatant, the suspension was plated on non-gelatinized plates and incubated for 30 min at 37°C. The remaining cell suspension was diluted to 1×10^6^ cells/100 uL on ice.

### Generation of Transgenic Mice

Female ICR mice were superovulated through consecutive injections of pregnant mare serum gonadotrophin (5 IU) and human chorionic gonadotropin (5 IU) every 48 hours before mating with fertile males. The females were screened for vaginal plugs the following morning (0.5 dpc, day post coitum). At 3.5 dpc, blastocysts were collected from mouse oviducts and cultured in a droplet of potassium simplex optimization medium, overlaid with embryo-tested mineral oil under a 5% CO_2_ humidified atmospheres at 37°C. The cultured blastocysts were then injected with 10 to 15 mES cells by piezo micromaniplulation and the reconstructed embryos were transferred into the uterus of 2.5 dpc pseudopregnant mothers. Chimerism was assessed using coat color, and foreign gene integration was detected via PCR amplification of genomic DNA from the chimeric mice. The chimeric mice were mated with wild-type ICR mice to obtain germ-line transmission offsprings.

### PCR and Southern Blot Analysis

For southern blotting, 20 ug of mouse genomic DNA from the tail biopsy were digested with the HindIII for 20 hours and were electrophoresed in a 1% agarose gel. After alkali denaturation, the digested genomic DNA was transferred onto a positively charged nylon membrane (Pharmacia USA) for 20 hrs. Prehybridization was performed for 2 hrs at 60°C. The probes were boiled for 10 min, and then placed immediately on ice. Hybridization was done overnight at 60°C. Alkaline phosphatase–conjugated anti-digoxigenin antibodies (1∶5000, Abcam) were added to the membrane after washing, and 300 uL of NBT/BCIP (Mylab Corporation DIGD-110) was added for staining. Positive controls containing different copy numbers of the brazzein plasmid were used to estimate the copy number of the transgene in the transgenic mice by comparing their band densities. The southern blot membrane was analyzed using the UN-SCAN-IT gel 6.1 software. We also used the following method for determination of the copy number of the transgene. The Primers Br (Forward) and Br (Reversal) were used to amplify a 520 bp fragment using mouse genomic from the tail as template. The 520 bp region from the Beta-casein intron to the brazzein gene was also used as the probe in the southern blot analysis. Wild-type mouse genomic DNA was used as the negative control and the pBC1-Br-loxP-neo-loxP vector DNA with the same amount wild-type mouse genomic DNA was used as the positive control. We calculated the copy number of the transgene using this formulary mass: copy number of transgene = N bp transgene DNA X1 microgram genomic DNA/3×10^9^ bp genomic DNA to figure out the concentration of the positive plasmid. Each sample was loaded with 20 µg genomic DNA and the concentrations of the plasmid were estimated as follows: 1 copy number is *equal* to 0.15 ng or 5 copies to 0.75 ng DNA and so on.

### Detection of Transcription of Recombinant Brazzein in the Mammary Gland Tissues

Total RNA extracted from various tissues, including liver, heart, spleen, lung, kidney, pancreas, uterus, and mammary glands of transgenic mice using Trizol reagent (Invitrogen, Carlsbad, CA, USA). The extracted RNA was then used as a template to synthesize cDNA using a first strand cDNA synthesis kit. A 170 bp PCR product was generated using the primers Brs: 5′-CTGGCTCTGGCTGACAAGTGTAAG-3′ and Bra: 5′-GTACTCGCAATAGTCGCAGATGC-3′ to verify the presence of the brazzein gene. Amplification was done with 30 cycles of 94°C for 30 s, 60°C for 30 s, and 72°C for 1 min. The mouse housekeeping gene glyceraldehyde-3-phosphate dehydrogenase (GAPDH) was used as the loading control with primers GA1 (5′GGTGAAGGTCGGTGTGAACG-3′) and GA2 (5′CTCGCTCCTGGAAGAGGTG-3′). RNA extracted from the mammary gland tissues of a non-transgenic mouse, with and without M-MuLV reverse transcriptase, was separately used as negative controls.

### Real-time PCR

Total RNA was isolated from transgenic mouse mammary gland tissues using Trizol (Invitrogen, Carlsbad, CA, USA). Complementary DNA was synthesized from 1 ug (20 uL reaction) of total RNA using a DNA eraser with the first strand cDNA Synthesis Kit for RT-PCR (TaKaRa, Japan). The mRNA expression was analyzed via qRT-PCR using the Brs and Bra primers. The expression level of the brazzein gene was compared with that of the housekeeping gene GAPDH. The cDNAs were amplified for 39 cycles using CHROMO4 MJ (Bio-Rad) with SYBR Green dye via a two-step amplification scheme (95°C for 15 s and 60°C for 30 s). The non-transgenic mouse sample was used as the negative control. Blank control templates devoid of reverse transcriptase were run in parallel. All samples were run in triplicate. After amplification, melting curves were constructed to confirm amplicon identity by increasing the temperature by 40°C at 0.5°C increments every 15 seconds. The data were analyzed using the ΔΔCt method [14].

### Enzyme-linked Immunosorbent Assay (ELISA)

Briefly, the mice were milked [15], the milk were then diluted with two volumes of PBS, and centrifuged at 4°C for 15 min at 8,000×g to separate the whey, casein, and fat fractions. The ELISA were performed as previously described [16]. The whey samples were diluted 1∶80 and the brazzein protein expression levels were determined using anti-brazzein specific antibody, generated by Abmart, Shanghai, China using the brazzein peptide. To determine the level of brazzein in the milk, a brazzein standard curve was established using duplicate measurements of the brazzein standard solution (Abmart, Shanghai, China), which were generated by spiking different amount of brazzein into cow milk. The brazzein level in milk was then determined using specific brazzein antibody in an ELISA and was measured at 450 nm using a Synergy™ HT Multi-Mode Plate Reader (BioTek Instruments Inc., Vermont, USA). The brazzein levels in the milk samples from transgenic or wild type mice were then obtained by calculating the ELISA results from the brazzein standard curve. Values are reported as measured concentrations.

### SDS-PAGE and Western Blots

Rabbit anti-brazzein polyclonal primary antibody (1∶200) and goat anti-rabbit IgG (1∶6000, GGHL-15A, ICL Lab) secondary antibody were used in the western blot analysis. To access the expression of the brazzein in transfected HEK-293 cell, cell culture supernatant was diluted in 1×SDS sample buffer (62.6 mM Tris-HCl, pH 6.8, 2% SDS, 10% glycerol, and 0.01% bromophenol blue) and incubated at 100°C for 5 min. To access the brazzein in the milk, the milk sample from the transgenic and wild type mice was diluted 10-fold with distilled water. The proteins in the milk samples and cell supernatant were separated via SDS-PAGE and transferred electrophoretically onto a polyvinylidene difluoride membrane. The immunoblots were incubated overnight with the primary polyclonal rabbit anti-brazzein antibody (1∶200, Abmart, China) at 4°C followed by incubation with secondary antibody (1∶6000 titer for goat anti-rabbit IgG, R&D, USA) for 1 h at room temperature. The brazzein proteins were detected using a Supersignal West PicoTrial enhanced chemiluminescence kit (Thermo).

### Immunofluorescence

The HEK-293 cells were fixed with 4% paraformaldehyde (PFA), and then washed three times with PBS. Cells were permeabilized and blocked with PBS, 0.1% Triton X-100, and 1 mg/mL bovine serum albumin. Primary anti-brazzein antibodies (1∶100) were then added in the blocking buffer and incubated overnight at 4°C. Cells were washed three times with PBS, a secondary goat anti-rabbit IgG at 1∶1000 (Alexa 488, Invitrogen, Carlsbad, CA, USA) was added for 1 hour at room temperature before the final washes in PBS. Transgenic and non-transgenic mammary gland tissues were fixed using 4% PFA, and the paraffin-embedded specimens were used for fluorescent immunohistochemical analysis. Brazzein protein was detected using goat anti-rabbit IgG (H+L) at 1∶200 (Alexa 488, Invitrogen) after overnight incubation with primary antibody at 1∶100 at 4°C.

### Sweetness Intensity Testing

The mice milk was collected as previous reported [17]. Briefly, 0.1 ml (2 IU) of oxytocin was injected by intraperitoneally using a 27-guage needle attached to a 1 ml syringe, within 1 minute, milk was aspired using a pipette through the opening of mammary gland. The boiled mouse milk was subject to a double-blind taste test with 14 volunteer participants consisting of six females and eight males (ages 18 to 60 years). None of the participants exhibited any irregularities in their sense of taste. All participants were randomly selected and given full disclosure of the aim of the study and their roles. The tasting protocol was designed according to the standard for Measuring Sensations of Taste [18]. Sucrose solutions at 0%, 0.6%, 2%, 4%, 6%, and 10% (w/v) were prepared as positive controls and the milk of wild-type mice as the negative control. Sample of mouse milk at 50 µl were dropped onto the anterior part of the tongue. The subject’s mouth was rinsed three times by drinking water after each test. The order of presentation of the samples was randomized and the taste test for each sample was repeated three times. The score was determined based on feedback categorized under “not sweet” (0), “uncertain whether sweetness was tasted” (0.5), “faintly sweet” (1.0), “sweet” (2.0), “very sweet” (3.0) and “extremely sweet” (4.0). The average score for 2% and 4% sucrose was established at 1.0 or 2.0, respectively. Sweetness scores were first evaluated with repeated measurement ANOVA (analysis of variance) followed by one-way ANOVA of the scores for different variants. A P values <0.05 were considered significant.

## Results

### Expression of Brazzein in HEK-293 Cell Lines

**Figure 2:**
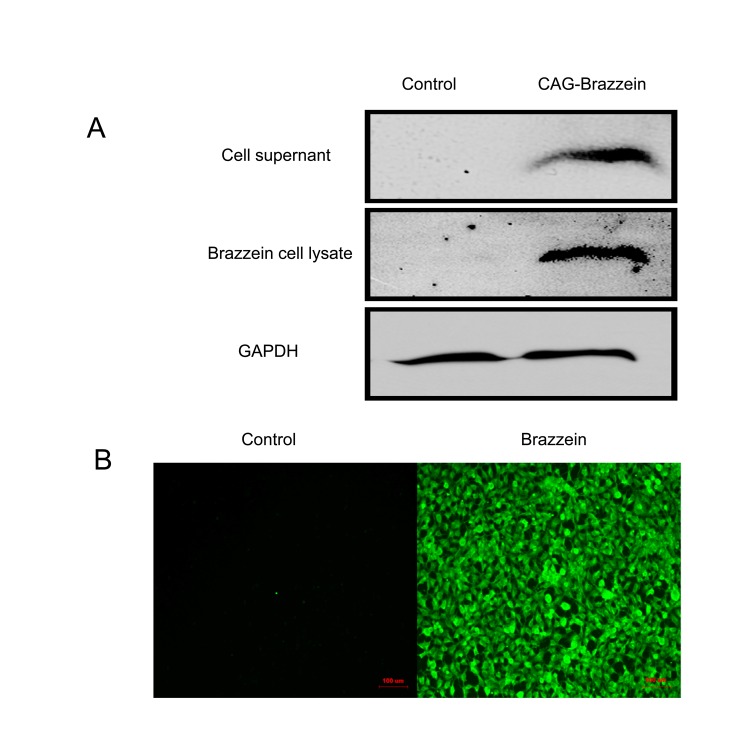
Expression of brazzein in HEK-293 cell line. (A) Western blot analysis of the lysate and supernatant of HEK-293 cells transfected with pCAG-Br-neo; the lysate and supernatant of HEK-293 cells transfected with pCAG-neo were used as the negative control. (B) Immunofluorescence assay of the lysate and supernatant of HEK-293 cells transfected with pCAG-neo and pCAG-Br-neo. Scale bars = 100 um.

To determine whether the brazzein gene can be expressed in mammalian cells, the pCAG-Br-neo vector (Figure 1A) and pCAG-neo vector were individually transfected into HEK-293 cells using Lipofectamine 2000 (Invitrogen, Carlsbad, CA, USA). The transgenes were expressed under the control of the cytomegalovirus enhancer and chicken Beta-actin (CAG) promoter. The cells were analyzed with an immunofluorescence assay 48 hours after transfection. Brazzein expression was detected in the cells transfected with pCAG-Br-neo, but not in those transfected with pCAG-neo (Figure 2B). Simultaneously, the cell culture medium was also collected and used for western blot analysis. As expected, Brazzein was detected in the medium of cells transfected with pCAG-Br-neo, but not in the medium of cells transfected with pCAG-neo (Figure 2A). These results indicate that brazzein can be expressed in mammalian cells and secreted by the cells.

### Establishment of Transgenic mES Cell Lines Containing a Mammary Gland–specific Brazzein Expression Vector

**Figure 3:**
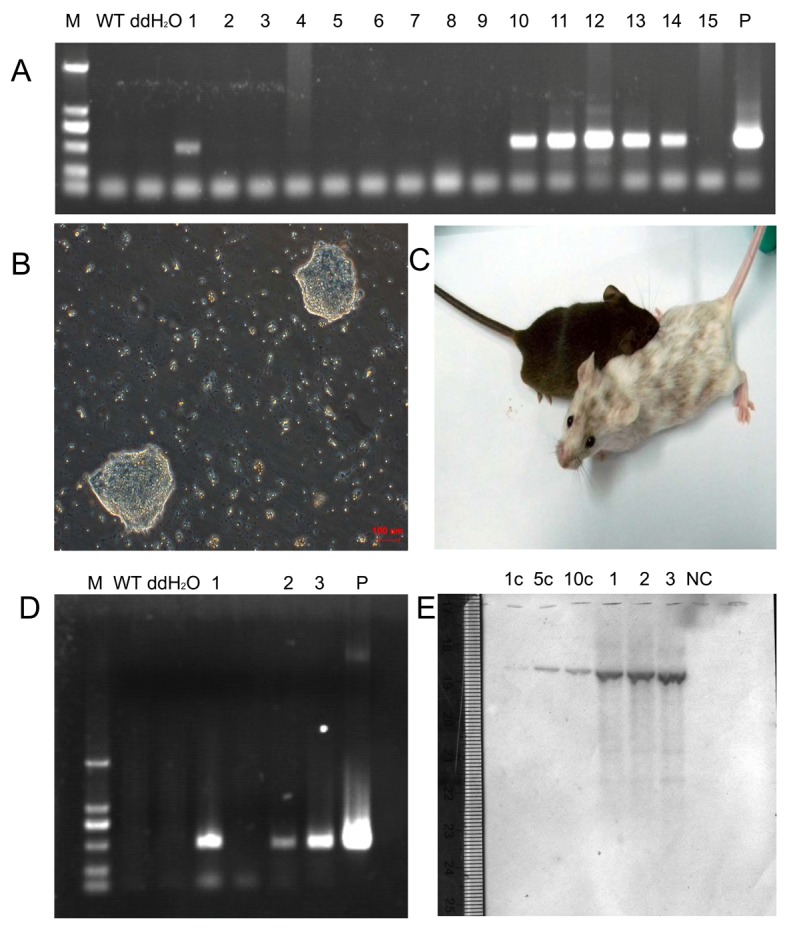
Identification of transgenic mice via PCR and Southern blot analysis. (A) PCR analysis of brazzein in mES cells. WT, non-transfected cells, ddH2O were used as templates in the negative controls; 1 to 15: genomic DNA from selected stable cell clones as templates; P = positive control of brazzein gene amplified via PCR using the pBC1-Br-loxP-neo-loxP vector as the template. (B) Screening of stable transgenic mES cell clones using G418 selection method. (C) Germline chimeric mouse and the offspring of chimeras. (D) PCR amplification of genomic DNA from transgenic mice. M is the DNA ladder; WT is the non-transgenic mouse, and P is the positive control. Numbers 1, 2 and 3 are different brazzein transgenic mice. (E) Southern blot analysis of mouse genomic DNA from the tails: Genomic DNA was isolated from wild-type and transgenic mice (1, 2, and 3), then digested with HindIII. The digested DNA were resolved on a DNA gel and blotted to a nitrocellulose member. The membrane was subjected to hybridization with the brazzein probe. NC, a non-transgenic mouse control, 1, 2 and 3, the transgenic mouse line; positive controls were done with 1, 5, or 10 copy numbers.

The pBC1-Br-loxP-neo-loxP DNA was transfected into mES cells, which was established in our laboratory from an embryo resulted from crossing of an Oct4-GFP mouse and a 129 mouse. Transfected cells were treated with 300 ug/mL G418 (Merck, Germany) for 7 days to select cells harboring the brazzein transgene. The transgenic cells maintained normal growth patterns and formed cell colonies with typical ES morphology (Figure 3B). Fifteen colonies were selected for individual propagation in 200 ug/mL G418. The integration of brazzein gene into each cell line was verified with PCR amplification of genomic DNA from the transfected cells, wherein a non-transfected mES genome was used as the negative control, and pBC1-Br-loxP-neo-loxP DNA as the positive control. Six brazzein-positive cell lines with the desired 520 bp PCR product were identified (Figure 3 A).

### Generation of Transgenic Mice

Three transgenic cell lines, No. 11, No. 12, and No. 13, were used as donors for generating chimeric mice through microinjection into blastocysts. After embryo transfer, six chimeric mice from the No. 11 cell line, three from the No. 12, and five from No. 13 were successfully produced. After mating with ICR mice, three transgenic mouse lines, designated as line 1, line 2, and line3 were established (Figure 3C). The desired 520 bp PCR product was detected using genomic DNA from the transgenic mice (Figure 3D). No sequence similarity to the wild-type mouse counterpart was found through homologous BLAST analysis. Southern blot analysis showed that digestion of the mouse gDNA with *Hind*III produced a 4.73 kb band (Figure 3E). The Southern blot membrane was analyzed using the UN-SCAN-IT gel 6.1 software, which showed that mouse line 1 had 37 transgenic copies of brazzein cDNA, mouse line 2 had 41 transgenic copies, and mouse line 3 had 46 transgenic copies.

### Brazzein mRNA Transcription in Mammary Glands and Other Tissues

**Figure 4:**
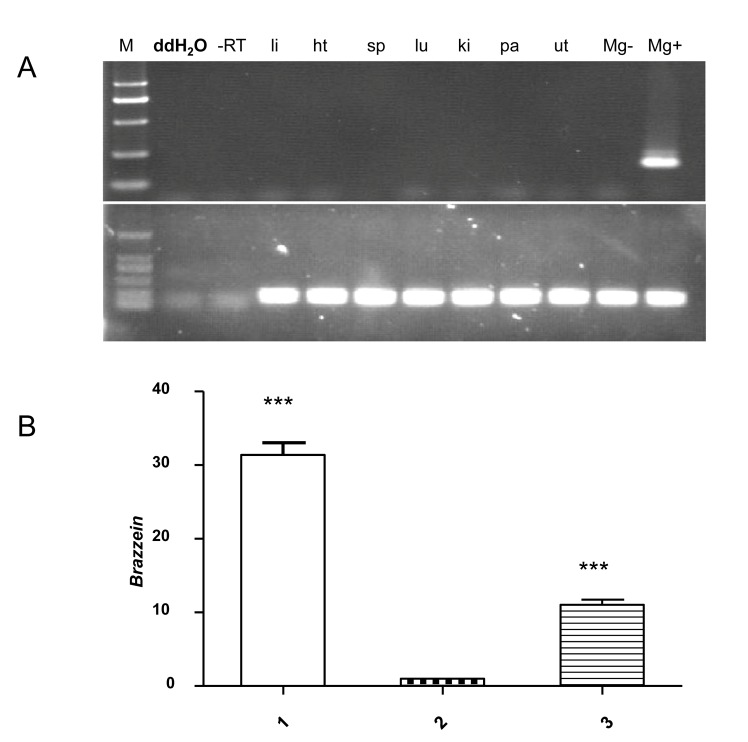
RT-PCR analysis of transgenic mouse from various tissues to determine relative transcripts levels of brazzein. (A) RT-PCR was performed to determine the tissue specificity and expression levels of brazzein in transgenic mice. The tissues analyzed were from liver (li), heart (ht), spleen (sp), lungs (lu), kidneys (ki), pancreas (pa), uterus (ut), and mammary glands (mg). M represents Marker; -RT represents no M-MuLV Reverse Transcriptase in the reaction to rule out genomic DNA contamination. The bottom panel is the result of the RT-PCR analysis of the mouse GAPDH gene. (B) The relative transcripts of brazzein in mammary gland tissues detected by real-time PCR were much higher than in other tissues. Total mRNA was extracted from mammary tissues of mouse lines 1, 2, and 3.The results are shown as means ± S.D. ***P<0.001 compared with control.

Brazzein gene transcription was determined via RT-PCR analysis of RNA from different tissues (liver, heart, spleen, lung, kidney, pancreas, uterus, and mammary glands) using primers Brs and Bra. Mouse GAPDH was used as the control for the reverse-transcribed RNA. Distilled deionized water, non-transgenic mammary tissue and samples without reverse transcription were used as the negative controls. Transcripts of the recombinant brazzein gene were not detected in the liver, heart, spleen, lung, kidney, pancreas, and uterus of all three transgenic mouse lines, but only in the mammary gland tissues from all three mouse lines (Figure 4A). This finding demonstrates the high tissue-specificity of brazzein gene transcription, with no leaky expression. The mRNA transcripts level in the mammary glands of mouse line 1 was much higher than those of mouse lines 2 and 3 (Figure 4B).

### Expression of Brazzein Sweet Protein in the Mammary Glands of Transgenic Mice

**Figure 5:**
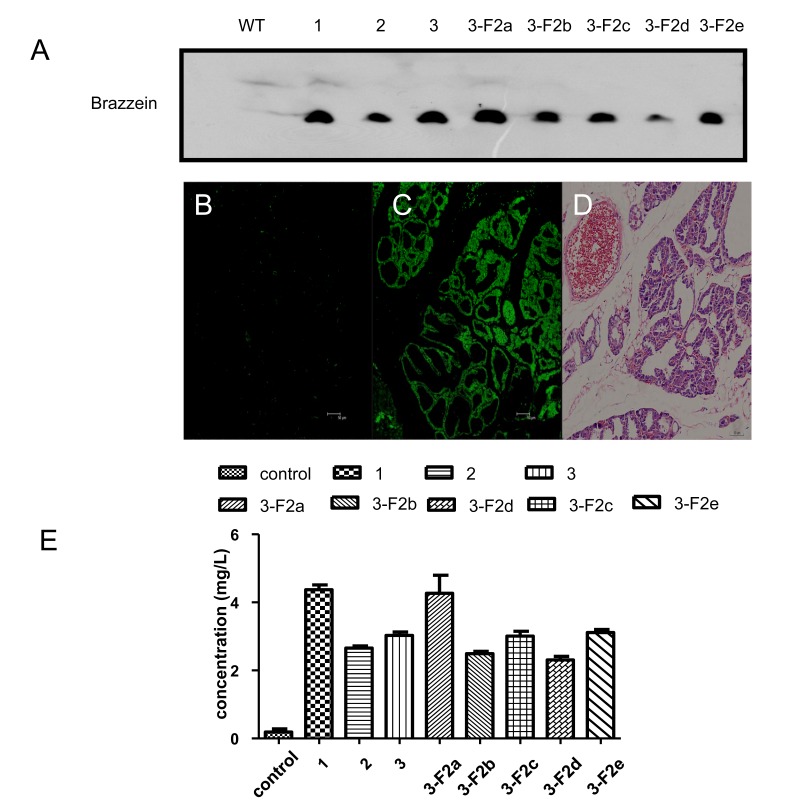
Detection of brazzein in the milk of transgenic mice. (A) Expression of brazzein in the mammary glands of transgenic mice under western blot analysis. Skim milk samples (1 uL each) were separated by SDS-PAGE and immunoblotted with anti-brazzein antibodies; WT, non-transgenic milk; 1, 2, and 3, milk from transgenic mice; 3-F2a to 3-F2e represent the milk produced by the F2 generation of mouse line (B) Immunofluorescent histochemical stains of brazzein in two sections of the mammary gland tissues. Non-transgenic mammary gland tissues were used as the negative control. Scale bars = 50 um. (C) Transgenic mouse mammary gland tissues. Scale bars = 50 um. (D) Hematoxylin and eosin staining of transgenic mammary gland tissues. Scale bars = 50 um. (E) Brazzein concentrations in the milk of different mice. Control, the milk of a non-transgenic mouse; 1, 2, 3, 3-F2a, 3-F2b, 3-F2c, 3-F2d, and 3-F2e are milk samples from the different transgenic mice.

Brazzein protein was detected in the milk of the three parent transgenic mouse lines and their progeny by western blot analysis (Figure 5A), whereas no corresponding signal was detected in the non-transgenic milk. The brazzein concentration was then quantified using an ELISA. The levels of brazzein in the milk of the three different male funder mice were as follows: line 1, 4.37 mg/L; line 2, 2.65 mg/L; and line 3, 3.026 mg/L. The brazzein expression levels in the milk from five different F2 progeny of mouse line 3 were as follows: 3-F2a, 4.26 mg/L; 3-F2b, 2.49 mg/L; 3-F2c, 3.00 mg/L; 3-F2d 2.30 mg/L; and 3-F2e, 3.11 mg/L (Figure 5E). These result show that the brazzein gene is specifically expressed in the mammary gland and that the transgene acquired a germline transmission.

We also performed an immunofluorescence assay to detect the brazzein expression in the paraffin-embedded mammary tissues. The mammary tissue of non-transgenic mice was used as the negative control (Figure 5B). As shown in Figure 5C, brazzein is widely expressed in the mammary tissue of a transgenic mouse, but not in that of the wild type mouse. Hematoxylin and eosin staining (Figure 5D) revealed that the transgenic mammary tissue structure is normal. In addition, the transgenic mice, mother and offsprings, lived a normal lifespan, which indicated that the offspring could be fed on brazzein-containing milk.

### Sweetness Analysis of Brazzein in Transgenic Mouse Milk

**Figure 6:**
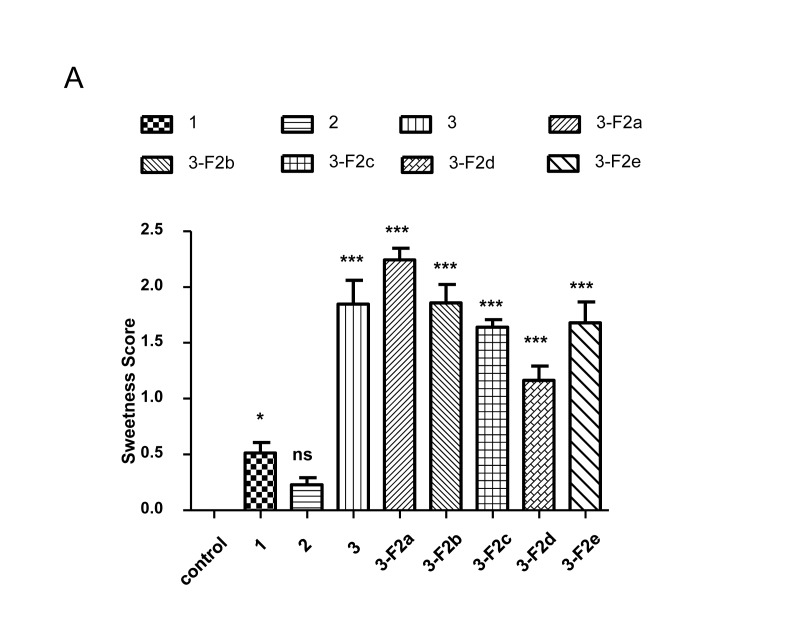
Sweetness test of recombinant brazzein. (A) Sweetness was scored from 1 to 4 as follows: (0) not sweet, (0.5) uncertain if sweetness was tasted, (1.0) faintly sweet, (2.0) sweet, (3.0) very sweet and (4.0) extremely sweet. Results of psychophysical experiments with brazzein are shown; data were averaged for the 14 volunteers. Error bars represent SD. Column patterns indicate different levels of sweetness compared with the control (non-transgenic milk); the others are the sweetness of milk from the different transgenic mice. The results are shown as means ± S.D. ***P<0.001 compared with control.

To determine whether the brazzein in transgenic mouse milk retained its sweetness, the milk was subject to sensory evaluation. The milk from three different non-transgenic mice was used as the negative control. To test the thermal stability of brazzein in the transgenic mouse milk, the milk was heated at 100°C for 5 min. The sweetness of the milk from mouse line 3 was comparable to 4.0% sucrose solution (Figure 6) and is significantly differed from that of the control milk (P<0.05). A similar sweetness was observed in the milk from offspring of mouse line 3 (Figure 6). The milk from mouse lines 1 and 2 were less sweet (Figure 6). The sweetness of the milk from mouse line 1 was comparable with that of a 0.5% sucrose solution.

## Discussion

In this study, we demonstrated that the plant gene, which encodes the sweet protein brazzein, can be actively expressed in transgenic mouse milk and that the recombinant brazzein protein retains palatable sweetness. The evidence for our conclusion comes from three different experiments. First, we detected the specific expression of the brazzein transcript in the mammary gland from transgenic mice. Second, western blotting, immunohistochemistry, and ELISA using brazzein specific antibody also showed the expression of the brazzein protein in the mouse mammary gland tissue specifically. Third, sweetness taste assay showed that the milk from the brazzein transgenic mice is significantly sweeter than the milk from nontransgenic mice.

To express and increase the sweetness of the transgenic brazzein in mammalian mammary gland, we modified the transgene by removing the first glutamic acid in brazzein, which could confer about twice the sweetness of regular brazzein [19]. We also changed the 29th amino acid from aspartic acid to lysine and the 41th from glutamic acid to lysine, which could also significantly increase the sweetness of brazzein [11]. Considering brazzein is a plant protein, it might not be properly expressed in animal cells. To overcome this problem, the gene sequence was optimized according to codon usage to allow optimal brazzein expression in animal cells [20]. We used the goat secretion signal Beta-casein signaling peptide to facilitate brazzein secretion into the milk [21], [22]. The Cre/loxP system was used to remove selective genes in transgenic animal [23]–[25], which would help prevent expressing an antibiotic with milk in future studies on large transgenic animals.

In a previous study, brazzein was expressed in *E. coli* fused with nuclease-ovomucoid for purification purposes. Fusion tags, irrespective of size, can potentially interfere with folding, function, or crystallization capabilities of a protein. Thus, the brazzein fusion protein had to be cleaved at the CNBr active sites to remove the fusion tag to maintain the sweetness [4]. Similarly, in a yeast expression system (*Saccharomyces cerevisiae*), the recombinant brazzein was fused with a glutathione S transferase tag for purification, which also needs to be removed to maintain sweetness [8]. In our mammary gland expression system, since milk can be consumed directly without purification, tags were unnecessary in the recombinant protein. Thus, further modification of the recombinant brazzein may not be needed while sweetness is efficiently conferred to the milk.

The various expression levels of brazzein protein in different mouse lines might be due to random integration of the transgene, which leads to positional effects on its expression [26]. Transgenes are often inserted into the transcriptionally inactive region of a chromosome, which results in low expression level [27]. Accordingly, in our three transgenic mouse lines that were derived from three different transgenic mES cells, the brazzein transcription levels were different, though their brazzein copy numbers are almost the same. Previous experiments have shown that foreign proteins can be specifically expressed at high levels in the mammary glands of transgenic mice [28]. Detectable levels could even reach one gram per liter. Although the brazzein expression levels in our mouse milk were not as high as previously reported, the brazzein protein expression enabled the milk to have a high level of sweetness. The sweetness rating score of the milk from mouse line 3, with 3.03 ug/mL brazzein protein, is comparable to that of 4% sucrose solution. This implies that our recombinant brazzein is nearly 1.3×10^4^ times sweeter than sucrose by weight.

The hematoxylin and eosin staining results showed that the tissues from the transgenic mice have normal morphologies as compared with nontransgenic mice. All parental mouse lines and their offspring were healthy and live normally, which also produced F2 generations. The mouse pups were routinely fed by their mothers with milk containing the brazzein protein, and their growth is as normal as the wild type littermates. This fact indicates that insertion of plant brazzein gene into mouse genome and its expression do not confer toxicity or other negative effects on the mice.

Although we were unable to determine of the content of the milk in the transgenic mice due to the limited amount of mouse milk, Zhanget et al [29] compared the proteome and nutrient composition of the colostrum and mature milk from three lines of transgenic cloned cattles, which express human alpha-lactalbumin, lactoferrin or lysozyme in the mammary gland, with those from cloned non-transgenic and conventionally bred normal animals. They did not find significant differences in milk among these groups and confirmed that the expression of a transgene does not affect the composition of milk.

Sweet-tasting brazzein milk could be used as food additive and does not require prior purification, a procedure similar to pharmaceutical proteins expressed in mammary glands, such as tissue-plasminogen activator [30] and human coagulation factor IX [31]. Thus, the processing cost and production time are tremendously reduced, which significantly favor market development.

In conclusion, our study verified that the plant gene brazzein could be actively produced in the milk of transgenic animals. The successful production of sweet milk in the mouse model provides novel and easier ways to produce important plant proteins in mammary glands. When applied to transgenic cattle or goats, their milk could be a good source for mass-producing brazzein, allowing the large-scale commercial production of this healthy alternative sweetener.
